# Factors affecting the quality of life of gastric cancer survivors

**DOI:** 10.1007/s00520-021-06683-y

**Published:** 2022-01-01

**Authors:** Jahyun Choi, Sanghee Kim, Mona Choi, Woo Jin Hyung

**Affiliations:** 1 Department of Nursing, Graduate School, Yonsei University, Seoul, South Korea; 2 College of Nursing & Mo-Im Kim Nursing Research Institute, Yonsei University, 50-1 Yonsei-ro, Seodaemun-gu, 03722 Seoul, South Korea; 3 Department of Surgery, College of Medicine, Yonsei University, Seoul, South Korea

**Keywords:** Stomach neoplasm, Cancer survivors, Survivorship, Quality of life

## Abstract

**Background:**

The number of gastric cancer survivors has been increasing, and such survivors experience various changes in their lives post-recovery. Adapting to these changes requires appropriate interventions that can improve their quality of life (QoL). This study was conducted to investigate the factors affecting the QoL of gastric cancer survivors and provide basic data for effective intervention.

**Methods:**

Data were collected between September 8 and September 29, 2017, from the Gastric Cancer Center at a tertiary hospital. Questionnaire surveys were conducted using the EORTC QLQ-C30/STO22, Self-Efficacy-Scale, Multidimensional Scale of Perceived Social Support, and Quality of Life-Cancer Survivors Questionnaire on gastric cancer survivors who were followed up for 3 years after gastrectomy. Data were analyzed using descriptive statistics, *t* test, ANOVA, Pearson’s correlation coefficient, and multiple regression analysis.

**Results:**

A total of 136 gastric cancer survivors completed the questionnaire survey. There were significant positive correlations of QoL with self-efficacy, functional status, and social support (*r* = .35, *p* < .001; *r* = .53, *p* < .001; *r* = .26, *p* < .001, respectively). There were significant negative correlations of QoL with general symptoms (*r* =  − .39, *p* < .001) and gastric cancer-specific symptoms (*r* =  − .51, *p* < .001). The regression model explained 48.3% of the QoL, and the affecting factors were gastric cancer-specific symptoms (*β* =  − .397, *p* < .001), religious belief (*β* = .299, *p* < .001), functional status (*β* = .251, *p* = .003), and self-efficacy (*β* = .191, *p* = .004).

**Conclusion:**

This study confirmed that gastric cancer-specific symptoms, spiritual well-being, self-efficacy, and functional status affect the QoL of gastric cancer survivors. Hence, these factors should be considered in the interventions to improve the QoL of gastric cancer survivors.

## Introduction

Gastric cancer is the fifth most common cancer globally [1]. In South Korea, gastric cancer has the highest rate of occurrence in terms of cancer [2]. This is associated with the high rate of *Helicobacter pylori* infection, damping dietary habits, and consumption of spicy foods [2, 3]. The 5-year relative survival rate of gastric cancer has increased from 43.9% in 1993–1995 to 76.5% in 2013–2017 in South Korea [4]. Three out of every four patients diagnosed with gastric cancer are estimated to survive for 5 years or more. Given that the number of gastric cancer survivors is expected to rise, it is necessary to pay more attention to their life after treatment.

Cancer survivors experience different physiological, psychological, functional, and social changes post-recovery [5–7]. In particular, gastric cancer survivors lose their gastric storage function after gastrectomy, followed by various physiological changes depending on the type of vagotomy and reconstruction, which can lead to weight loss and iron and vitamin B_12_ absorption disorders [8]. Most patients improve over time or manage their diet, but in some patients, the quality of life (QoL) may be severely degraded [9]. When they adapt well to the aforementioned changes, they are able to live a healthy life and improve their QoL [10]. Hence, they need interventions that aid their adaptation to multiple aspects of their lives post-recovery.

Most studies on cancer survivors have been conducted on survivors of breast cancer and childhood cancer [11–14]. Even in South Korea, there has not been much research on gastric cancer survivors despite the high occurrence rate of this cancer and high survival rate of individuals with this cancer. Moreover, relevant studies on gastric cancer survivors have focused on solitary aspects, such as the relationship between a type of gastrectomy and the QoL [15, 16], relationship between nutritional status and the QoL [17–19], and occupations and their influencing factors [20]. The lack of comprehensive research warranted an analysis of how multiple aspects affect the adaptation and QoL of gastric cancer survivors in various ways.

The purpose of this study was to determine gastric cancer survivors’ symptoms, self-efficacy, functional status, social support, and QoL and analyze the factors affecting QoL. The study attempted to provide fundamental data for the implementation of effective interventions to improve gastric cancer survivors’ adaptation and QoL.

## Methods

### Study design

This study used a descriptive cross-sectional design to investigate the factors affecting the QoL of gastric cancer survivors. This study was approved by the institutional review board (IRB no.4–2017-0651) of Severance Hospital in Seoul, South Korea.

### Participants

The inclusion criteria of the study were as follows: (1) gastric cancer survivors who completed their cancer treatment such as gastrectomy and chemotherapy; (2) ≥ 19 years old; (3) no evidence of recurrence or metastasis on medical record; and (4) absence of any other major health problems requiring treatment.

The number of subjects required for this study was calculated using G Power 3.1.9 programs. When the effective size of multiple regression analysis (f ^2^) was 0.15, significance level (α) was 0.05, the number of predictors was 12, and the power (1-β) was 0.80; the number of subjects was calculated as 127. The target number of subjects considering a 10% drop-out rate was 140. Of the 140 patients initially recruited, the data of four patients were excluded as they did not complete the survey. Finally, data for 136 survivors were included in the analysis record.

### Procedure

The data were collected between September 8 and September 29, 2017, from the Gastric Cancer Center at Severance Hospital in Seoul. The purpose and procedure of the study were explained to the medical staff, and their cooperation was requested for data collection. Patients with whom follow-ups were conducted for over 3 years after gastrectomy were informed about the study. The researchers explained the purpose of the study to the subjects that met the inclusion criteria, and the survey was conducted after obtaining the written consent of patients. The subjects completed a self-administered questionnaire that took around 20 to 30 min to complete. The disease-related characteristics were confirmed by the researchers after the survey from the hospital’s electronic medical record.

### Measures

#### Symptoms

The symptom scales of the Korean version of the European Organization for Research and Treatment of Cancer Quality of Life Questionnaire-Core 36 (EORTC QLQ-C30) and EORTC QLQ the gastric cancer module (EORTC QLQ-STO22) were used to measure symptom level [21, 22]. The symptom scales of EORTC QLQ-C30 and EORTC STO-22 have 13 and 22 items, respectively. The raw scores were transformed to scores ranging from 0 to 100 according to the scoring manual [23]. A higher score on a symptom scale indicated a higher level of symptom. In this study, Cronbach’s α values of the symptom scales of EORTC QLQ-C30 and EORTC QLQ-STO22 were 0.74 and 0.83, respectively.

#### Self-efficacy

To measure self-efficacy, the Korean version of the self-efficacy scale was used [24]. It consists of 13 items rated on a five-point Likert scale ranging from 1 (disagree strongly) to 5 (agree strongly), with the scoring of negative items reversed. A higher score indicated greater self-efficacy. Cronbach’s α was 0.83 in a previous study [25] and 0.85 in this study.

#### Functional status

Functional status was measured using the function scale of the Korean version of the EORTC QLQ-C30 [21]. This scale consists of 15 items, and its raw scores are transformed to scores ranging from 0 to 100 according to the scoring manual [23]. A higher score indicated a higher level of functional status. The Cronbach’s α was 0.84 in this study.

#### Social support

The multidimensional scale of perceived social support (MSPSS) was used to measure social support [26]. The MSPSS includes 12 items representing the support of three domains (family, friends, significant others). The domain of support from significant others refers to support provided by meaningful others, which, in this study, included the support provided by the medical staff such as doctors and nurses. The original scale is seven-point Likert scale; however, we employed a five-point Likert scale ranging from 1 (disagree strongly) to 5 (agree strongly), based on a previous study [27]. A higher score indicated a higher level of social support. Cronbach’s α was 0.83 in a previous [27] and this study.

#### Quality of life

QoL was measured using the Korean version of the quality of life-cancer survivors questionnaire (QOL-CS) [28]. The QOL-CS includes 41 items representing the four domains of physical, social, psychological, and spiritual well-being. The items are rated on a four-point Likert scale ranging from 1 (not at all) to 4 (very much). The scoring of negative items was reversed, and a higher score represented a better QoL. Cronbach’s α was 0.83 in this study.

### Statistical analysis

Statistical analysis was performed using SPSS version 25.0. The demographic and disease-related characteristics of the participants were analyzed using frequencies, percentages, means, and standard deviations. Symptoms, self-efficacy, functional status, social support, and QoL were analyzed using means and standard deviations. Differences in symptoms, self-efficacy, functional status, social support, and QoL according to the demographic and disease-related characteristics of the participants were analyzed using *t* tests, ANOVA, and post-hoc analysis, which was performed by the Scheffe test through the Levene’s test, and confirmed that homoscedasticity was satisfied. The correlations among symptoms, self-efficacy, functional status, social support, and QoL of the participants were analyzed using Pearson’s correlation coefficient. To identify factors affecting the QoL of the participants, a multiple regression analysis was performed. A *p*-value of < 0.05 was considered to be statistically significant.

## Results

The sociodemographic and disease-related characteristics of the participants are shown in Table 1. Men accounted for 62.5% of the participants. The mean age of the participants was 58.8 ± 10.8 years. Of the participants, 85.3% were married, 59.0% were employed, and 52.9% had religious beliefs. The spouse was the main caregiver for 71.3% of the participants. Most of the participants were diagnosed at the first stage of cancer (76.5%), and the mean duration after gastrectomy was 5.1 ± 2.3 years. Moreover, 77.2% of the participants had not received chemotherapy.

**Table 1:** Characteristics of the participants

Variable	Category	n(%) (N = 136)
Sex	Male	85(62.5)
	Female	51(37.5)
Age (yr.)	<65	88(64.7)
	≥65	48(35.3)
Marital status	Married	116(85.3)
	Unmarried (single, divorced, bereaved)	20(14.7)
Occupational status	Employed	79(59.0)
	Unemployed	55(41.0)
Religious belief	Yes	72(52.9)
	No	64(47.1)
Main caregiver	Spouse	97(71.3)
	Non-spouse	39(28.7)
Cancer stage (TNM)	I	104(76.5)
	II	18(13.2)
	III	14(10.3)
Duration after gastrectomy (yr.)	≤3<4	50(36.8)
	≤4<5	46(33.8)
	≥5	40(29.4)
Chemotherapy	Yes	31(22.8)
	No	105(77.2)
Body mass index (kg/m^2^) ^†^	<18.5	15(11.0)
	≤18.5<23	107(78.7)
	≤23<25	14(10.3)

^†^ < 18.5 (underweight), ≤ 18.5 < 23 (normal), ≤ 23 < 25 (overweight)

The symptoms, self-efficacy, functional status, social support, and QoL of the participants are shown in Table 2. The mean general symptom score was 17.40 ± 10.55 (range, 0–100), and the most common general symptoms were fatigue (31.54 ± 18.46), diarrhea (26.23 ± 25.14), and insomnia (20.59 ± 28.99). The mean gastric cancer-specific symptom score was 18.07 ± 10.64 (range, 0–100), and the most common gastric cancer-specific symptoms were anxiety (29.49 ± 22.07), body image (25.00 ± 30.29), and dry mouth (22.30 ± 25.68). The mean self-efficacy score was 3.74 ± 0.66 (range, 1–5). The mean functional status score was 85.56 ± 11.31 (range, 0–100), and role functioning (89.95 ± 15.49) was found to be more robust than the other functions. The mean social support score was 3.53 ± 0.61 (range 1–5), and family support (4.34 ± 0.77) was found to be greater than support from the other two domains. Finally, the mean QoL score was 3.01 ± 0.28 (range 1–4).

**Table 2:** Symptoms, self-efficacy, functional status, social support, and quality of life of the participants

Variable	Category	Mean ± SD (*N* = 136)
General symptoms ^a^		17.40±10.55
(EORTC QLQ-C30)	Fatigue	31.54±18.46
	Diarrhea	26.23±25.14
	Insomnia	20.59±28.99
	General pain	13.48±19.41
	Appetite loss	12.25±21.78
	Financial difficulties	11.76±18.38
	Constipation	10.54±18.01
	Dyspnea	9.56±18.55
	Nausea and vomiting	6.86±12.74
Gastric cancer-specific symptoms ^a^		18.07±10.64
(EORTC QLQ-STO22)	Anxiety	29.49±22.07
	Body image	25.00±30.29
	Dry mouth	22.30±25.68
	Eating restrictions	19.53±17.40
	Gastric-related pain	16.36±15.19
	Reflux symptoms	16.26±17.37
	Hair loss	15.07±22.94
	Dysphagia	8.17±9.96
	Taste	6.86±15.24
Self-efficacy ^b^		3.74±0.66
Functional status ^a^		85.56±11.31
	Role functioning	89.95±15.49
	Social functioning	88.36±17.84
	Emotional functioning	86.21±16.92
	Physical functioning	83.82±13.27
	Cognitive functioning	81.37±18.04
Social support ^b^		3.53±0.61
	Family support	4.34±0.77
	Friend’s support	3.67±0.85
	Health care provider’s support	2.75±1.06
Quality of life ^c^		3.01±0.28
	Social concerns	3.49±0.39
	Physical well-being	3.47±0.34
	Psychological well-being	2.85±0.37
	Spiritual well-being	2.35±0.59

^a^Range in score from 0 to 100, ^b^5-point Likert scale, ^c^4-point Likert scale

The differences in the symptoms, self-efficacy, functional status, social support, and QoL according to participants characteristics are shown in Table 3. The gastric cancer-specific symptom score differed significantly according to age. In the post-hoc analysis, participants less than 65 years of age had a higher amount of gastric cancer-specific symptoms than those over 65 years of age. The self-efficacy score was significantly lower among those aged over 65 years, unemployed, not having any religious beliefs, and having a non-spouse as the main caregiver. The functional status score was significantly higher among those who were employed, and the social support score was significantly higher among those who spouse was their main caregiver. The QoL score was significantly higher among those having religious beliefs, diagnosed at the first stage of cancer, and not having received chemotherapy.

**Table 3:** Symptoms, self-efficacy, functional status, social support, and quality of life according to participant characteristics (N = 136)

Variables	General symptoms		Gastric cancer- specific symptoms		Self-efficacy		Functional status		Social support		Quality of life	
	M±SD	t/F(p)	M±SD	t/F(p)	M±SD	t/F(p)	M±SD	t/F(p)	M±SD	t/F(p)	M±SD	t/F(p)
Sex												
Male	56.60±36.04	-.86 (.39)	17.84±11.21	-.34 (.74)	3.82±0.61	1.89 (.06)	85.96±12.81	.60 (.55)	3.50±0.60	-.94 (.35)	3.01±0.25	.05 (.96)
Female	62.27±38.62		18.47±9.72		3.60±0.73		84.88±8.29		3.60±0.63		3.01±0.33	
Age (yr.)												
<65	18.01±10.00	.91 (.37)	19.89±11.20	2.98 (.004)*	3.87±0.59	3.14 (.002)*	85.86±10.61	.42 (.67)	3.59±0.58	1.41 (.16)	2.98±0.30	-1.79 (.08)
≥65	16.29±11.53		14.74±8.68		3.51±0.72		85.00±12.58		3.44±0.65		3.07±0.24	
Marital status												
Married	59.53±36.75	-.61 (.54)	18.03±10.82	.13 (.90)	3.74±0.68	-.09 (.93)	85.57±11.17	-.05 (.96)	3.57±0.61	-1.62 (.11)	3.02±0.28	-.83 (.41)
Unmarried	54.04±38.98		18.36±9.79		3.73±0.49		85.44±12.35		3.33±0.57		2.96±0.30	
Occupational status												
Employed	55.47±34.70	1.36 (.18)	17.62±9.93	.70 (.49)	3.93±0.51	-4.07 (<.001)**	87.54±10.89	-2.48 (.01)*	3.56±0.57	-.29 (.77)	3.04±0.25	-1.34 (.18)
Unemployed	64.29±39.99		18.93±11.62		3.45±0.75		82.71±11.34		3.53±0.65		2.97±0.32	
Religious belief												
Yes	58.12±33.23	.20 (.84)	18.96±10.98	-1.02 (.31)	3.85±0.58	-2.18 (.03)*	86.08±9.66	-.57 (.57)	3.63±0.63	-1.95 (.05)	3.10±0.27	-4.09 (<.001)**
No	59.40±41.07		17.08±10.24		3.61±0.73		84.97±12.96		3.43±0.57		2.91±0.26	
Main caregiver												
Spouse	60.05±36.27	-.66 (.51)	18.37±11.09	-.52 (.61)	3.87±0.60	-3.65 (<.001)**	86.03±11.81	-.76 (.45)	3.60±0.58	-2.01 (.04)*	3.04±0.28	-1.68 (.09)
Non-Spouse	55.43±39.03		17.33±9.52		3.43±0.70		84.39±10.00		3.37±0.65		2.95±0.29	
Cancer stage (TMN)												
I	58.25±37.44	.55 (.58)	17.49±10.84	1.79 (.17)	3.77±0.62	.57 (.56)	85.98±11.39	.37 (.69)	3.57±0.59	1.13 (.33)	3.04±0.28	3.82 (.02)* a>b
II	66.14±37.67		22.44±9.02		3.60±0.74		83.58±10.14		3.34±0.62		2.85±0.27	
III	52.74±33.65		16.83±10.41		3.69±0.83		84.92±12.55		3.50±0.72		3.00±0.26	
Time since gastrectomy (yr.)												
≤3<4	17.59±10.39	.52 (.60)	18.05±11.53	.36 (.70)	3.79±0.65	.28 (.76)	85.60±12.06	.25 (.78)	3.58±0.51	1.24 (.29)	2.99±0.29	.51 (.60)
≤4<5	16.22±10.52		17.18±10.66		3.70±0.61		86.33±9.73		3.42±0.63		3.05±0.26	
≥5	18.53±10.92		19.13±9.58		3.72±0.74		84.61±12.20		3.61±0.68		3.00±0.31	
Chemotherapy												
Yes	57.29±36.41	.24 (.81)	19.62±10.45	-.92 (.36)	3.65±0.78	.76 (.45)	83.66±11.27	1.07 (.29)	3.40±0.66	1.46 (.15)	2.92±0.28	2.20 (.03)*
No	59.15±37.32		17.62±10.70		3.77±0.62		86.12±11.31		3.58±0.59		3.04±0.28	
Body mass index (kg/m^2^)												
<18.5	20.17±10.25	.81 (.45)	20.14±9.46	.32 (.73)	3.83±0.62	.15 (.86)	85.19±9.64	.17 (.84)	3.57±0.60	.12 (.88)	2.94±0.39	.88 (.42)
≤18.5<23	16.82±10.64		17.79±10.99		3.73±0.68		85.82±11.32		3.54±0.61		3.01±0.26	
≤23<25	18.86±10.34		18.04±9.45		3.75±0.58		83.97±13.40		3.46±0.65		3.08±0.29	

^*^p < .05, **p < .001

The correlations among the symptoms, self-efficacy, functional status, social support, and QoL are shown in Table 4. The QoL was significantly positively correlated with self-efficacy (*r* = 0.35, *p* < 0.001), functional status (*r* = 0.53, *p* < 0.001), and social support (*r* = 0.26, *p* < 0.001). In contrast, the QoL was significantly negatively correlated with general symptoms (*r* =  − 0.39, *p* < 0.001) and gastric cancer-specific symptoms (*r* =  − 0.51, *p* < 0.001).

**Table 4:** Correlations among symptoms, self-efficacy, functional status, social support, and quality of life (*N* = 136)

Variables	Symptoms		Self-efficacy	Functional status	Social support	Quality of life
	General symptoms	Gastric cancer- specific symptoms				
	r(p)					
Symptom						
General symptoms	1					
Gastric cancer-specific symptoms	.60** (<.001)	1				
Self-efficacy	-.17* (.04)	-.08 (.35)	1			
Functional status	-.58** (<.001)	-.54** (<.001)	.23* (.01)	1		
Social support	-.16 (.06)	-.06 (.46)	.17* (.04)	.29** (<.001)	1	
Quality of life	-.39** (<.001)	-.51** (<.001)	.35** (<.001)	.53** (<.001)	.26** (.001)	1

^*^p < .05, **p < .001

To identify factors affecting the participants’ QoL, religious belief, cancer stage, exposure to chemotherapy, among the sociodemographic and disease-related characteristics, and the general symptoms, gastric cancer-specific symptoms, self-efficacy, functional status, and social support, which showed a significant correlation with QoL, were entered to perform a multiple regression analysis. Of these, the categorical variables, i.e., religious belief and exposure to chemotherapy, were treated as dummy variables; we included a group without religious beliefs and a group that was not exposed to chemotherapy.

When the Durbin-Watson correlation coefficient was checked to verify the basic assumptions of the regression analysis on the QoL, there was no autocorrelation with the coefficient 1.85. When the tolerance limit and the variance inflation factor for the multicollinearity test were measured, the tolerance limit was 0.32 ~ 0.91, which was higher than 0.1, and the dispersion expansion factor was 1.10 ~ 3.13, which was less than 10, confirming that there was no problem in terms of multicollinearity.

Through a multiple regression analysis, it was determined that the model was significant (*F* = 16.79,* p* =  < 0.001) and showed 48.3% of variance. The variable of gastric cancer-specific symptoms (*β* =  − 0.397, *p* < 0.001) was the strongest predictor, followed by religious belief (*β* = 0.299, *p* < 0.001), functional status (*β* = 0.251, *p* = 0.003), and self-efficacy (*β* = 0.191, *p* = 0.004). These had an effect on the QoL, in the order listed (Table 5).

**Table 5:** Factors affecting quality of life by multiple regression analysis (*N* = 136)

Variables	Categories	Β	SE	*β*	t	*p*
(Constant)		2.136	.224		9.517	< .001
Gastric cancer-specific symptoms		− .010	.002	− .397	− 4.768	< .001
Religious belief	Yes (ref: No)	.168	.036	.299	4.611	< .001
Functional status		.006	.002	.251	3.005	.003
Self-efficacy		.081	.028	.191	2.936	.004
Social support		.035	.031	.076	1.148	.253
Exposure to chemotherapy	Yes (ref: No)	− .044	.073	-.065	-.595	.553
General symptoms		.001	.002	.042	.500	.618
Cancer stage		.002	.046	.005	.043	.966
*R*^2^ = .514, Adj. *R*^2^ = .483, *F* = 16.792, *p* < .001						

*B* unstandardized beta; *SE* standard error; Adj. *R*^2^ adjusted *R*^2^

## Discussion

Of the general symptoms of gastric cancer, the most common symptoms among survivors who participated in this study were fatigue, diarrhea, and sleep disorder. Of the specific symptoms of gastric cancer, the most common symptoms were anxiety, physical changes, and dryness of mouth. This result is consistent with previous studies [16, 29]. Gastric cancer survivors continue to experience main symptoms; however, the degree of symptoms experienced by survivors differs based on the time elapsed since gastrectomy [19]. Many studies have revealed that one of the most common symptoms experienced by cancer survivors, regardless of the type of cancer, is fatigue [11, 30–32]. Fatigue is a sense of severe physical and emotional exhaustion. In this study, the fatigue score was 31.54 ± 18.46, which exceeds the cut-off score 30 suggested by clinical guideline [33]. This is similar to the score of gastric cancer survivors 1 year after gastrectomy (28.1 ± 17.9) [19] and that of gastric cancer survivors who have aged more than 5 years (24.4 ± 19.8) [34]. The degree of symptoms gradually decreases over time; however, fatigue is the highest level among the symptoms they experience and persists. Fatigue impedes cancer survivors from taking on roles and activities to an extent that greatly affects their daily activities and lowers their QoL. Therefore, providing appropriate intervention to address the problem of cancer survivors’ fatigue is crucial [31]. Additionally, cancer survivors continuously experience psychological symptoms such as anxiety, along with physical problems, due to the changes in their lives [35]. After treatment, it is important to manage gastric cancer survivors’ symptoms to enable their adaptation to their new life and improve their QoL. In this regard, intervention programs such as complex exercise therapy and health education therapy, which have produced successful results with survivors of other types of cancer, may be effective [12, 36].

The self-efficacy of gastric cancer survivors in this study was higher than the efficacy levels reported in previous studies conducted on gastric cancer patients and breast cancer patients who underwent chemotherapy and radiotherapy, respectively. [37, 38]. The participants in this study experienced either extended or permanent survivorship after passing through phases of acute survivorship during their cancer treatment; thus, they had a high possibility of not being afflicted with cancer post-treatment. The high level of self-efficacy of these survivors may also have been a result of the trust and self-confidence they developed through the experience of the disease and its treatment [39]. When cancer survivors enter extended survivorship after passing acute survivorship, they want to return to the personal and social roles they were performing before by managing the symptoms caused by the side effects of the treatment [40]. If their self-efficacy is high during this phase, they are able to adapt to changes in diverse aspects of their lives, and gain control over their lives [39, 40]. Therefore, to improve gastric cancer survivors’ adaptation to changes and their QoL, interventions to maintain and promote self-efficacy become necessary. Hence, intervention programs such as voluntary service activity, cognitive behavior treatment, and meditation may be effective [41, 42].

The functional status of gastric cancer survivors who participated in this study was as high as the levels reported in previous studies on long-term gastric cancer survivors [30, 34]. Among the subcategories of functional status, cognitive function had the lowest and role function had the highest score; this result was consistent with that of previous studies [30, 34]. A study found that chemotherapy provided to gastric cancer patients affected their cognitive function [43]. However, in the current study, 77.2% of the survivors did not undergo chemotherapy. Cognitive function could have been the lowest among all functions due to other causes such as the conventional aging process rather than the cancer treatment [44]. Although role function was found to be at the highest level among all the subcategories of functional status, 41.0% of survivors took a leave of absence or quit their jobs. Cancer survivors’ return to their jobs and household labor after the end of treatment is important to them and their families [7]. Difficulty in returning to the role function they had before their cancer diagnosis negatively affects their QoL [45]. Therefore, providing interventions that focus on their return to the society is important. Providing interventions before their functional status is lowered makes it easier for them to return to society. In terms of intervention plans, education about cancer symptoms and symptom management methods is necessary to reduce the adverse after-effects of cancer treatment and increase the survivors’ possibility of engaging in social activities. To ensure that gastric cancer survivors receive timely information on job return and the occupational rehabilitation program, they must be offered consultations on occupational rehabilitation or be connected with a team of occupational rehabilitation professionals [46].

The level of social support available to gastric cancer survivors in this study as well as the extent of support offered from various domains—with family ranking first, followed by friend and medical staff—was similar to results on social support in a previous study on breast cancer survivors [47]. Family ranked first on the most amount of support received by survivors because spouses accounted for 71.3% of the caregivers. Gastric cancer survivors in this study received the lowest amount of social support from the medical staff. Cancer survivors continue to experience physical and psychological symptoms after the end of cancer treatment [14]; hence, receiving support from one’s surroundings as well as receiving the appropriate resources, information. and emotional support from someone who is going through cancer journey with them is necessary. Therefore, even after the end of cancer treatment, the development of an intervention program that can provide support and information from medical staff through periodic evaluation should be considered.

The QoL of the participants in this study was higher than the QoL reported in previous studies on thyroid cancer survivors, colon cancer survivors, and lymphoma survivors [28, 48]. Gastric cancer survivors are likely to have a relatively higher QoL than survivors of other cancer types because of the regular medical checkups implemented in South Korea. These checkups have allowed for the detection of cancer in its initial stages, and its consequent treatment, in over 50% cases of gastric cancer [49]. However to maintain this higher QoL, effective intervention may be needed.

A study reported that cancer survivors’ participation in spiritual activities and their spiritual well-being positively affected their adaptation to life and QoL after cancer diagnosis [50]. In this study, participants who reported following a religion had a significantly higher QoL than those who did not. However, it was hard to analyze whether a religion was related to one’s participation in spiritual activities and thereby improved spiritual well-being. Therefore, it will be necessary to undertake further research to explore these relationships.

With regard to the disease characteristics of gastric cancer survivors, those whose cancer was in the first stage, and those who did not undergo chemotherapy had a significantly higher QoL. According to a previous study, gastric cancer survivors who did not undergo chemotherapy had a higher QoL [16]. On the contrary, some studies have reported that exposure to chemotherapy did not affect gastric cancer survivors’ QoL [51]. The current study’s overall results on QoL may have been affected by the fact that 76.5% of the participants were diagnosed at the first stage of cancer. Therefore, for accurate comparison, it will be necessary to conduct repeated research that includes cancer survivors diagnosed at later stage of cancer and those who received chemotherapy.

In this study, following a religion was found to affect the QoL of gastric cancer survivors positively. Moreover, specific symptoms of gastric cancer, self-efficacy, and functional status affected their QoL. However, social support did not affect gastric cancer survivors’ QoL. The reason for the result could be that the 76.5% of participants were diagnosed at the first stage of cancer and 77.2% did not receive chemotherapy. This meant that participants were able to care for themselves and were less dependent on others for help or support. Moreover, given that 71.3% of participants had spouses as caregivers, and that the level of family support was highest among all the subcategories of social support, it would be helpful to analyze aspects of social support available to gastric cancer survivors using tool measuring matrimonial interdependence. Furthermore, cancer survivors may not be able to ask for help or support from their surroundings because they also experience fear of stigma or shame around them [52], which will require further study.

This study has some limitations. It employed convenience sampling, wherein a small number of long-term gastric cancer survivors were recruited from just one tertiary hospital Seoul, South Korea. Thus, the results of the study may not be generalizable to all gastric cancer survivors. Additionally, since the study was cross-sectional, it could not appropriately analyze the causal relationships between variables; these relationships must be analyzed carefully in future study.

Nevertheless, this study was novel in its inclusion of participants who were gastric cancer survivors demonstrating a high occurrence rate and high survival rate among most cancer survivors. This study contributes to the existing literature by analyzing the factors that affect gastric cancer survivors’ QoL in diverse ways.

## Conclusions

Our results indicate that gastric cancer-specific symptoms, religious belief, functional status, and self-efficacy are significant factors affecting the QoL of gastric cancer survivors. These factors should be considered in the development of interventions to improve the QoL in gastric cancer survivors.

## Data Availability

Authors confirmed that some access restrictions apply to the data underlying the findings. This study used self-administered questionnaire and disease-related characteristics were collected from the hospital electronic medical record. Therefore, the data from this study could not be shared publicly due to confidentiality issues. If any inquiry, please contact the corresponding author (sangheekim@yuhs.ac).

## References

[1] The Global Cancer Observatory. Stomach cancer. http://gco.iarc.fr/today/data/factsheets/cancers/7-Stomach-fact-sheet.pdf. Accessed 10 Jul 2020.

[2] National Cancer Information Center. Status of cancer prevalence. https://www.cancer.go.kr/lay1/S1T654C655/contents.do. Accessed 20 Jun 2020.

[3] Song MLH, Kang D (2015). Epidemiology and screening of gastric cancer in Korea. J Korean Med Assoc.

[4] National Cancer Information Center. 5 year cancer survival rate. https://www.cancer.go.kr/lay1/S1T648C650/contents.do. Accessed 20 Jun 2020.

[5] Carrillo GM, Santamaría NP (2019). Life after a gastrectomy: experience of patients with gastric cancer. Enferm Clin.

[6] Brinkman TM, Recklitis CJ, Michel G, Grootenhuis MA, Klosky JL (2018). Psychological symptoms, social outcomes, socioeconomic attainment, and health behaviors among survivors of childhood cancer: current state of the literature. J Clin Oncol.

[7] Yamauchi H, Nakagawa C, Fukuda T (2017). Social impacts of the work loss in cancer survivors. Breast Cancer.

[8] Lim CHKS, Kim WC, Kim JS, Cho YK, Park JM (2012). Anemia after gastrectomy for early gastric cancer: long-term follow-up observational study. World J Gastroenterol.

[9] Cable CTCC, Showalter T, Ahluwalia R, Song J, Whitfield P (2011). Prevalence of anemia after Roux-en-Y gastric bypass surgery: what is the right number?. Surg Obes Relat Dis.

[10] Naus MJ, Ishler MD, Parrott CE, Kovacs SA (2009). Cancer survivor adaptation model: conceptualizing cancer as a chronic illness. J Clin Psychol.

[11] Pope Z, Lee JE, Zeng N, Lee HY, Gao Z (2019). Feasibility of smartphone application and social media intervention on breast cancer survivors’ health outcomes. Transl Behav Med.

[12] Dangi AA, Aurangabadkar SK, Deo MV (2018). Effect of a structured yoga program on fatigue, depression, cardiorespiratory fitness, and quality of life in a postmenopausal breast cancer survivor. Int J Yoga.

[13] Brinkman TM, Li C, Vannatta K, Marchak JG, Lai JS, Prasad PK (2016). Behavioral, social, and emotional symptom comorbidities and profiles in adolescent survivors of childhood cancer: a report from the childhood cancer survivor study. J Clin Oncol.

[14] Cheng H, Sit JW, Chan CW, So WK, Choi KC, Cheng KK (2013). Social support and quality of life among Chinese breast cancer survivors: findings from a mixed methods study. Euro J Oncol Nurs.

[15] Kwon OK, Yu B, Park KB, Park JY, Lee SS, Chung HY (2020). Advantages of distal subtotal gastrectomy over total gastrectomy in the quality of life of long-term gastric cancer survivors. J Gastric Cancer.

[16] Lee SS, Chung HY, Kwon OK, Yu W (2016). Long-term quality of life after distal subtotal and total gastrectomy: symptom-and behavior-oriented consequences. Ann Surg.

[17] Tanaka C, Kanda M, Murotani K, Yoshikawa T, Cho H, Ito Y (2019). Long-term quality of life and nutrition status of the aboral pouch reconstruction after total gastrectomy for gastric cancer: a prospective multicenter observational study (CCOG1505). Gastric Cancer.

[18] Lee SS, Yu W, Chung HY, Kwon OK, Lee WK (2017). Using quality of life scales with nutritional relevance after gastrectomy: a challenge for providing personalized treatment. J Gastric Cancer.

[19] Kim AR, Cho J, Hsu YJ, Choi MG, Noh JH, Sohn TS (2012). Changes of quality of life in gastric cancer patients after curative resection: a longitudinal cohort study in Korea. Ann Surg.

[20] Rottenberg Y, Jacobs JM, Ratzon NZ, Grinshpun A, Cohen M, Uziely B (2017). Unemployment risk 2 years and 4 years following gastric cancer diagnosis: a population-based study. J Cancer Surviv.

[21] Aaronson NK, Ahmedzai S, Bergman B, Bullinger M, Cull A, Duez NJ (1993). The European organization for research and treatment of cancer QLQ-C30: a quality-of-life instrument for use in international clinical trials in oncology. J Natl Cancer Inst.

[22] Vickery CW, Blazeby JM, Conroy T, Arraras J, Sezer O, Koller M (2001). Development of an EORTC disease-specific quality of life module for use in patients with gastric cancer. Euro J Cancer.

[23] Fayers PM, Aaronson NK, Bjordal K, Groenvold M, Curran D, Bottomley A (2001). The EORTC QLQ-C30 scoring manual.

[24] Sherer M, Maddux JE, Mercandante B, Prenticc DS, Jacobs B, Rogers RW (1982). The self-efficacy scale: construction and validation. Psychol Rep.

[25] Oh PJ, Lee EO, Tae YS, Um DC (1997). Effects of a program to promoto self-efficacy and hope on the self-care behaviors and the quality of life in patinets with leukemia. J Korean Acad Nurs.

[26] Zimet GD, Dahlem NW, Zimet SG, Farley GK (1988). The multidimensional scale of perceived social support. J Pers Assess.

[27] Shin JS, Lee YB (1999). A study on the influence of social support on the psychosocial wellbeing of the unemployed. J Korean Soc Welf.

[28] Cho J, Kang D, Kim IR, Kim WS, Ferrell B, Kim SJ (2017). Validation of the Korean version of the quality of life-cancer survivors (QOL-CS-K) questionnaire in lymphoma survivors. Cancer Res Treat.

[29] Yu W, Park KB, Chung HY, Kwon OK, Lee SS (2016). Chronological changes of quality of life in long-term survivors after gastrectomy for gastric cancer. Cancer Res Treat.

[30] Park KB, Lee SS, Kwon OK, Chung HY, Yu W (2017). Chronological changes in quality of life after distal gastrectomy for gastric cancer. J Gastric Cancer.

[31] Berger AM, Mooney K, Alvarez-Perez A, Breitbart WS, Carpenter KM, Cella D (2015). Cancer-related fatigue, version 2. J Natl Compr Canc Netw.

[32] Zhang JK, Fang LL, Zhang DW, Jin Q, Wu XM, Liu JC (2016). Type D personality in gastric cancer survivors: association with poor quality of life, overall survival, and mental health. J Pain Symptom Manage.

[33] Snyder CF, Blackford AL, Okuyama T, Akechi T, Yamashita H, Toyama T (2013). Using the EORTC-QLQ-C30 in clinical practice for patient management: identifying scores requiring a clinician's attention. Qual Life Res.

[34] Yu W, Park KB, Chung HY, Kwon OK, Lee SS (2016). Chronological changes of quality of life in long-term survivors after gastrectomy for gastric cancer. Cancer Res Treat.

[35] Mitchell AJ, Ferguson DW, Gill J, Paul J, Symonds P (2013). Depression and anxiety in long-term cancer survivors compared with spouses and healthy controls: a systematic review and meta-analysis. Lancet Oncol.

[36] Liu L, He X, Feng L (2019) Exercise on quality of life and cancer-related fatigue for lymphoma survivors: a systematic review and meta-analysis. Support Care Cancer 27:4069–4082 10.1007/s00520-019-04983-y 31300873

[37] Ko HK, Park GJ (2011). Effects of self-efficacy promotion program on self-efficacy, self-care behavior, and quaity of life in breast cancer patients receiving radiotherapy. Asian Oncol Nurs.

[38] Kim JH (2012). Influencing factors on depression in stomach cancer patients receiving chemotherapy. J Korean Adult Nurs.

[39] Philip EJ, Merluzzi TV, Zhang Z, Heitzmann CA (2013). Depression and cancer survivorship: importance of coping self-efficacy in post-treatment survivors. Psychooncology.

[40] Hoffman AJ, von Eye A, Gift AG, Given BA, Given CW, Rothert M (2009). Testing a theoretical model of perceived self-efficacy for cancer-related fatigue self-management and optimal physical functional status. Nurs Res.

[41] Germino BB, Mishel MH, Crandell J, Porter L, Blyler D, Jenerette C, et al., editors (2013) Outcomes of an uncertainty management intervention in younger African American and Caucasian breast cancer survivors. Oncol Nurs Forum 40:82–92 10.1188/13.ONF.82-92 23269773

[42] Yi M, Cha J, Ryu Y (2014). Changes of self-efficacy, depression, and posttraumatic growth in survivors with breast cancer participating breast cancer prevention volunteering. Int J Contents.

[43] Oh PJ, Lee JR (2016). Effect of cancer symptoms and fatigue on chemotherapy-related cognitive impairment and depression in people with gastrointestinal cancer. J Korean Acad Nurs.

[44] Calman KC (1984). Quality of life in cancer patients-an hypothesis. J Med Ethics.

[45] Altice CK, Banegas MP, TuckerSeeley RD, Yabroff KR (2017) Financial hardships experienced by cancer survivors: a systematic review. J Natl Cancer Inst 109:djw205 10.1093/jnci/djw205 PMC6075571 27754926

[46] Leensen MC, Groeneveld IF, Van Der Heide I, Rejda T, Van Veldhoven PL, Van Berkel S (2017). Return to work of cancer patients after a multidisciplinary intervention including occupational counselling and physical exercise in cancer patients: a prospective study in the Netherlands. BMJ open.

[47] Seo EY, Kwon S (2018). The influence of spiritual well-being, self-esteem, and perceived social support on post traumatic growth among breast cancer survivors. Asian Oncol Nurs.

[48] Applewhite MK, James BC, Kaplan SP, Angelos P, Kaplan EL, Grogan RH (2016). Quality of life in thyroid cancer is similar to that of other cancers with worse survival. World J Surg.

[49] Kim JH, Kim SS, Lee JH, Jung DH, Cheung DY, Chung WC (2018). Early detection is important to reduce the economic burden of gastric cancer. J Gastric Cancer.

[50] Forouzi MA, Tirgari B, Safarizadeh MH, Jahani Y (2017). Spiritual needs and quality of life of patients with cancer. Indian J Palliat Care.

[51] Kassam Z, Mackay H, Buckley CA, Fung S, Pintile M, Kim J (2010). Evaluating the impact on quality of life of chemoradiation in gastric cancer. Curr Oncol.

[52] Fujisawa D, Umezawa S, Fujimori M, Miyashita M (2020). Prevalence and associated factors of perceived cancer-related stigma in Japanese cancer survivors. Jpn J Clin Oncol.

